# Applying after-action reviews to child and family teams to improve mental health service linkage within child welfare services: a study protocol

**DOI:** 10.1186/s43058-023-00479-3

**Published:** 2023-10-05

**Authors:** Marisa Sklar, Ryan Kenneally, Gregory A. Aarons, Danielle L. Fettes

**Affiliations:** 1 Department of Psychiatry, University of California, San Diego, 9500 Gilman Drive (0812), La Jolla, CA 92093-0812 USA; 2 ACTRI Dissemination and Implementation Science Center, University of California, San Diego, 9500 Gilman Drive, La Jolla, CA 92093 USA; 3 Child and Adolescent Services Research Center, 3665 Kearny Villa Rd., Suite 200N, San Diego, CA 92123 USA; 4 ImplementatioN Science and Team Effectiveness in Practice Children’s Mental Health Research Center, 3665 Kearny Villa Rd., Suite 200N, San Diego, CA 92123 USA

**Keywords:** Child welfare services, Shared decision-making, Team effectiveness research, Implementation science, After-action review, Debrief, Child family team

## Abstract

**Background:**

Half of child-welfare-involved children and adolescents meet the criteria for at least one mental health diagnosis. This project proposes to improve successful mental health service linkage in child welfare services (CWS) by adapting and testing the after-action review (AAR) team effectiveness intervention to augment the child and family team (CFT) services’ intervention. Despite being both required and a collaborative approach to service planning, CFT meetings are implemented with questionable fidelity and consistency, rarely including the voice of children and families as intended.

**Methods:**

Using a parallel group trial design, with non-equivalent comparison groups, and qualitative and quantitative methodology, this study will tailor and assess the impact of the AAR on enhancing CFT outcomes. The authors will conduct a qualitative needs assessment targeting the ongoing implementation of the CFT services intervention in a large, publicly funded, CWS system. A qualitative inquiry consisting of interviews and focus groups with key stakeholders will result in the preparation of an action plan to address identified gaps between the current and desired CFT services intervention outcomes. The AAR implementation strategy will be adapted and tailored to address the CFT services’ intervention needs. To test the effectiveness of the AAR on improving outcomes associated with the CFT services intervention, we will utilize blocked randomization of four CWS caseworkers from two CWS system regions to either the intervention condition (CFT + AAR) or standard implementation (CFT as usual). The authors will collect data from the CWS caseworkers and additional CFT members via web-based surveys. Mechanisms of the AAR team effectiveness intervention for CFT implementation will be assessed.

**Discussion:**

By inclusion of child and family voice, the AAR-enhanced CFT should lead to increased fidelity to the CFT intervention and greater levels of parental satisfaction with the service and shared decision-making, thus resulting in enhanced follow-through with service plans and linkage to mental health treatment services for children. The knowledge gained by this randomized clinical trial has the potential to benefit service delivery and integration for CWS leaders, caseworkers, formal and informal CFT member support persons, parents/caregivers, and children with open cases. Improving intervention effectiveness, both at the system and family levels, is crucial for practice efficiencies and improved child and family outcomes.

**Trial registration:**

NCT05629013. Approval date: November 28, 2022 (version 1).

**Trial sponsor:**

University of California, San Diego.

**Responsible party:**

Danielle Fettes.

Contributions to the literature

- Limited research in child welfare services (CWS) has focused on the team unit for facilitating successful and sustained child/family engagement in decision-making.
- Merges the fields of team effectiveness research and implementation science to enhance the child and family team (CFT) services’ intervention with an evidence-based team development intervention.
- Partnership between two key public sector service settings, CWS and specialty mental health, will facilitate an examination of whether the enhanced CFT meetings result in improved service linkage for children with mental health service needs.

## Child mental health in child welfare

Nearly 620,000 children and youth were victims of child abuse and neglect in 2018 [1]. The burden of child maltreatment includes negative social, emotional, behavioral, and health effects on child victims [2], as well as a fiscal impact. Recent estimates suggest the cost of child maltreatment in the USA, the majority of which results from confirmed cases of child neglect [1], exceeds $400 billion per year [3], a figure which excludes the costs of child welfare services (CWS) unsubstantiated investigations. The prevalence of mental health need is high among child welfare-involved children [4, 5]. Half of child-welfare-involved children and adolescents meet the criteria for at least one current mental disorder [6], the most common of which are disruptive disorder (27%) including conduct disorder (20%) and oppositional defiant disorder (12%), attention-deficit/hyperactivity disorder (11%), anxiety and depressive disorders (18% and 11%, respectively), and posttraumatic stress disorder (4%). Former foster youth report higher rates of mental health concerns and over one third of foster youth with need received no behavioral health services in the prior 12 months [7].

Child-welfare-involved children’s elevated rates of childhood trauma are directly associated with poor mental health and disrupted social development [8]. Evidence increasingly demonstrates the acute and long-term impact of adverse childhood experiences on child mental health, most recently showing the specific child maltreatment impacts in early adolescence, with higher depressive symptoms, trauma symptoms, anxiety symptoms, and externalizing problems [9].

## Family voice and shared decision-making in child welfare services

Engaging families in shared decision-making promotes safety, permanency, and physical and mental wellbeing of children and families in CWS systems and is central to successful service linkage [10]. Effective family engagement occurs when CWS practitioners actively collaborate and partner with family members throughout their involvement with the CWS system, empowering them in the process [10]. For parents receiving CWS, the timely completion of treatment is part of a specified Action Plan. Services can only be effective when clients fully participate, yet tension between collaboration and compliance in CWS is a challenge to meaningful family engagement.

Involving parents in treatment planning through shared decision-making is vital to child outcomes, with fewer child removals and fewer recurrences [11], thus resulting in decreased risk of child trauma, behavioral concerns, and mental health concerns. Yet, neither parents nor caseworkers perceive that Action Plans are created with mutual influence [12]. In fact, most parents believe they have no voice or input [13]. In further disenfranchisement of families, caseworkers often deprioritize their work with parents, focusing instead on mandated interactions to which they are held accountable (e.g., child visitations, court) [14]. Taken together, social work theory and training pay credence to the critical role of family engagement, voice, and decision-making in service plans, yet, in practice, the inclusion of families’ active voices toward the goal of shared decision-making remains largely absent.

## The promise of child and family team (CFT) meetings

Family meeting models such as child and family team (CFT) meetings in CWS are family-centered and collaborative ways to develop individualized, effective service plans based on mutual agreement [15]. Each child or youth is required to have a CFT meeting within 60 days of entering CWS to identify areas of behavioral, emotional, and/or social needs and complete an initial case Action Plan. Relying on teamwork approaches, CFT meetings intend to give children and families a voice in creating and guiding their Action Plans [16], facilitate shared decision-making, empower families to work with CWS agencies [17, 18], and address organizational need for teams to perform “complex, interdependent, dynamic, and ambiguous tasks [19]”. CFT meetings also promote positive outcomes for children—fewer children enter care, more are placed in residential settings [20], and improvement in youth problem behaviors, child functioning, child impairment, and mental health [16]. Parent engagement in CFT meetings is positively associated with permanency outcomes, including more placement in kinship care, placement stability, shorter stays in out-of-home placement, and increased family reunification.

Effective teamwork, required throughout the process of family meetings to ensure fidelity to the model and successful outcomes [15, 21], brings resources, knowledge, and services into family meetings to develop high-quality planning and implementation, which ultimately results in better outcomes for children and families [22]. Yet, in CWS CFT meetings, the quality of teamwork and proper adherence to the family-centered, collaborative view can be impaired by the inherent challenges of serving multiple needs and viewpoints that vary across team members, as well as the pressures and constraints of CWS.

## Shared mental models as a key tenet for team performance

One of the most fundamental challenges in the CFT meeting is parent active participation in shared decision-making, leading to coordinated Action Plans [14, 23]. For example, caseworkers often deprioritize parents’ voice and/or parents are unaware of their intended leadership role in CFT meetings [12, 13]. Because teamwork is not often optimized [15], CFT meetings may be less likely to adhere to the model and be ineffective in engaging parents and reaching a mutually agreed upon Action Plan, including mental health services utilization. One way to better optimize teamwork is to enhance the shared mental models of CFT members. Shared mental model theory posits that effective team performance on tasks requires members to have similar cognitive representations of task requirements, procedures, and members’ responsibilities [24, 25]. Strategies that generate clear shared mental models help team members create a more accurate shared view of a task by teaching each member about the roles and responsibilities of other team members [26]. Strategies that build similar [27], accurate, and shared mental models [28] could clarify and bolster parents’ role in the CFT meeting process.

## After-action reviews as a team implementation strategy

One strategy to enhance shared mental models that improve team performance is the after-action review (AAR). The AAR is an output-focused, team development intervention [29] used for improvement through individual and team-level reflection and learning. While other output-focused team development interventions exist, the AAR holds tremendous promise in enhancing CFT meetings as a team-based implementation strategy.

The goal of AARs is to have team members engage in an activity of reflection following the conclusion of a performance cycle or event [30]. AARs are considered a learning-based intervention since they focus on reflection of the outcome of a work period, and also the processes involved with reaching that outcome [29]. AARs are also optimal following a critical period of performance (such as the CFT meeting). Although originating in the US military to create “a state of mind where everybody is continuously assessing themselves, their units, and their organizations and asking how they can improve” [31], AARs have since been used across a variety of settings [30, 32, 33] to provide a forum for teams to learn, create, and implement plans to improve team interaction that can be applied to future scenarios and goals [32].

AARs allow team members to engage in collective sense-making after an event, overtly reflecting upon the processes and emergent states (e.g., shared mental models) experienced and associated outcomes, and developing strategies and goals for future events to improve upon future outcomes [30]. For example, AARs facilitate team performance via the emergent state of enhanced psychological safety [30, 34], an integral mechanism in which mutual respect and an atmosphere that is blame-free enable participants to feel safe sharing and discussing [35] and improved team processes, such as communication and coordination. When individual views are shared in a well-facilitated discussion, these perspectives can be supported, challenged, modified, and combined until some degree of consensus between team members is achieved [30]. By targeting team processes and emergent states, teams that employ AARs can improve their effectiveness by 25% [36], with downstream impacts to improved patient outcomes across medical settings [37, 38].

## Mechanistic model and how AARs will improve child/parent outcomes

This project aims to leverage and apply knowledge from the field of team effectiveness research to improve CWS child/family outcomes. Specifically, the proposed project will enhance the current CFT services intervention with an evidence-based team development intervention (i.e., AARs) to improve the mechanisms of team processes and team emergent states for CFT members (including CWS caseworkers and children/families), resulting in enhanced service/clinical outcomes (i.e., specialty mental health services linkage).

**Fig. 1:**
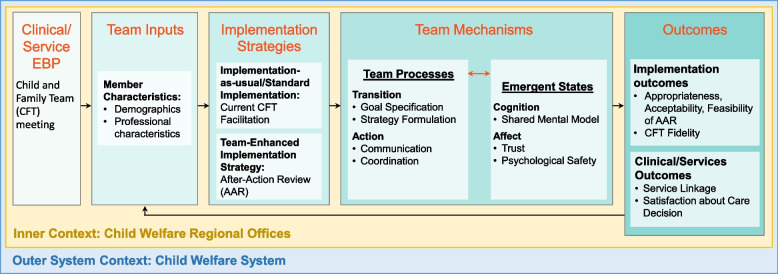
Mechanistic model to adapt and apply the after-action review to implementation of child and family team meetings

The AAR team development intervention, hereafter referred to as CFT + AAR, is hypothesized to improve outcomes associated with the CFT meeting services intervention through enhanced team processes and emergent states. See Fig. 1 for the proposed mechanistic model and Table 1 for the proposed measures for model constructs. The AAR aims to boost emergent states of stronger shared mental models amongst team members (Cognition), and greater levels of trust and psychological safety amongst team members (Affect). Additionally, the AAR intends to improve team processes of communication and coordination (Actions) and positive perceptions of the specified goal and formulated strategy for the CWS child/family Action Plan (Transition). By enhancing the mechanisms of emergent states and team processes, the AAR facilitates improved fidelity and child/family outcomes. Specifically, CFT meeting fidelity is exemplified when child/family voice and shared decision-making amongst team members is present. When CFT meeting fidelity is enhanced, greater satisfaction with the child/family Action Plan may be achieved, ultimately resulting in successful specialty mental health services linkage.

**Table 1:** Measures for model constructs by intervention group

Measure	Construct	CFT	CFT + AAR
**Input**			
Demographics	Team member characteristics	✓	✓
**Mechanisms**			
Working Alliance Inventory-Short Revised (WAI-SR) Goal	Team Processes Goal Specification	✓	✓
Working Alliance Inventory-Short Revised (WAI-SR) Task	Team Processes Strategy Formulation	✓	✓
Collaboration & Satisfaction About Care Decisions (CSACD)—Collaboration	Team Processes Communication and Coordination	✓	✓
Working Alliance Inventory-Short Revised (WAI-SR) Bond	Emergent States Trust	✓	✓
Card-Sorting Task	Emergent States Shared Mental Model	✓	✓
McAllister Affect-Based Trust	Emergent States Trust	✓	✓
Psychological Safety Climate Measure	Emergent States Psychological Safety	✓	✓
**Outcomes**			
Feasibility of Intervention Measure (FIM)	AAR Team Implementation Strategy Feasibility		✓
Acceptability of Intervention Measure (AIM)	AAR Team Implementation Strategy Acceptability		✓
Intervention Appropriateness Measure (IAM)	AAR Team Implementation Strategy Appropriateness		✓
Working Alliance Inventory-Short Revised (WAI-SR) Overall Score	CFT Clinical Intervention Fidelity Overall collaborative relationship	✓	✓
Parent Participation Engagement Measure (PPEM)	CFT Clinical Intervention Fidelity Collaboration and engagement	✓	✓
Collaboration & Satisfaction About Care Decisions (CSACD)—Satisfaction	CFT Clinical Intervention Outcome Satisfaction with decisions	✓	✓
SD County Behavioral Health Administrative Data	CFT Clinical Intervention Outcome Behavioral Health Service Linkage	✓	✓

Within team environments, shared mental models refer to the degree to which individual team members perceive key concepts (i.e., team, task) in a similar manner. For the CFT services intervention, shared mental models regarding perceptions of member roles, responsibilities, and goals can mediate more effective and efficient treatment planning. Team affect (i.e., trust and psychological safety) may also serve as mechanisms mediating success of the CFT services intervention. Psychological safety, a group-level phenomenon that correlates with team learning behaviors and performance, facilitates team members’ feelings of security [39] and focus on collective goals and problem prevention, rather than on self-protection [40–44]. Similarly, team trust is associated with decreased inefficiencies, greater negotiating effectiveness, and greater team effort, thus mediating overall team effectiveness [45–47]. Given the sensitivity and high stakes associated with CFT meetings (i.e., child placement, reunification), CFT members—particularly children/family—may feel uncomfortable fully participating for fear of negative repercussions. When CFT members experience enhanced shared mental models, psychological safety, and trust, they may be more open and willing to voice their opinions to collaborate on the treatment planning process for the CWS case.

The AAR also aims to result in high-quality team processes exemplified by greater communication and coordination (Actions), and more positive perceptions of the specified goal and formulated strategy for the CWS child/family case (Transition). Group psychological safety and trust can influence “speaking up, or voice” [48], or verbal communication among team members. This open communication is vital for the CFT services intervention to reach its desired outcome—shared decision-making among team members, including CWS children/families, with regard to goals and the Action Plan. By targeting these proximal team effects, the AAR team-based implementation strategy aims to enhance CFT services intervention through the inclusion of child/family voice and shared decision-making. By increasing fidelity to the CFT services intervention, CWS child and family members may exhibit greater levels of satisfaction with the Action Plan, and specifically improved linkage to needed specialty behavioral health services.

## Method

This study is part of a National Institute of Mental Health Advanced Laboratories for Accelerating the Reach and Impact of Treatments for Youth and Adults with Mental Illness Center [Center is P50 MH126231-01A1] that will implement a randomized control trial to develop and test team-based implementation strategies to improve services for children with mental health and developmental needs across service systems. This study aims to leverage and apply knowledge from team effectiveness research to improve outcomes associated with the services intervention currently used in CWS CFTs. Guided by the Exploration, Preparation, Implementation, Sustainment (EPIS) framework [49] and the Center’s Team Effectiveness for Implementation Science model (Fig. 1), this study aims to adapt and apply the team development intervention of the AAR to the implementation of the CFT meeting for a large, publicly funded, CWS system. Using a parallel group trial design, with non-equivalent comparison groups, and qualitative and quantitative methodology, this study will tailor and assess the impact of the AAR on enhancing CFT outcomes.

### Study objectives

#### Specific aim 1

Consistent with EPIS Exploration and Preparation phases, the research team will use qualitative methodology to explore current CFT clinical intervention implementation efforts in a large, publicly funded, county CWS system to better understand discrepancies that may exist between current and desired CFT services intervention outcomes. The needs assessment will occur over three phases.

Working in collaboration with County CWS, the first phase will center on defining the focus and scope of the needs assessment. Specifically, areas of concern regarding current CFT services’ intervention implementation efforts will be uncovered through informant interviews and focus groups with key stakeholders, including CWS leadership, supervisors, and front-line providers, as well as CWS-involved caregivers. Need indicators and/or data that have potential to confirm revealed concerns will be identified. Data sources to address and explore the magnitude of the uncovered concerns will be considered and prioritized.

The second phase will center on gathering and analysis of data. Specifically, this second phase will document the current status of CFT services’ intervention implementation efforts with regard to the concerns identified in phase one, including identification of mental health services linkage for children. Results from the data collected will be compared to the desired CFT services intervention outcomes. A list of need areas, wherein a discrepancy exists between current and desired CFT services intervention outcomes, will be compiled and factors that may be amenable to intervention will be identified.

The third phase of the need assessment aims to facilitate the preparation of an action plan to address the needs. The list of needs from phase two will be prioritized based on the magnitude of the discrepancy between current and desired outcomes, degree of difficulty in addressing the needs, and the potential consequences of neglecting the needs. Possible solutions to address the prioritized needs will be generated and examined. An action plan linking the needs with solutions will be prepared.

Interview and focus group data will be analyzed iteratively using a template organizing style [50]. Recordings will be professionally transcribed, checked for accuracy, and imported into NVivo software [51]. Transcripts will be read to gain familiarity with the content and to identify themes. NVivo will be used for line-by-line coding [52] using an a priori code list that was developed from theoretical and conceptual considerations and the initial reading of the transcripts. While the coding scheme will be formed along predetermined categories, it will remain flexible to accommodate new codes. Analytic processes will be reviewed by the Methods Core qualitative methods team.

#### Specific aim 2

Consistent with the EPIS Preparation phase, the research team will use the prepared action plan (Specific Aim 1) to generate an adapted and tailored AAR implementation strategy to address CFT services intervention needs. A collaborative process between the research team and CWS leadership resulted in the selection of the AAR as the team-based implementation strategy most appropriate for improving the CFT in CWS based upon its real-time applicability, the complexity of the “team”, and all team members being in a shared space.

Adaptations for the AAR will ensure maintenance to the core four questions AARs aim to answer [53]: (1) What was expected to happen?; (2) What actually occurred?; (3) What went well, and why?; and (4) What can be improved, and how? First, a general AAR tool based upon factors shown to be important for team effectiveness [54] that incorporates the twelve evidence-based practices for effective AARs [32] will be developed. The AAR tool will be further tailored and adapted to incorporate the needs prioritized through Specific aim 1. An iterative process with CWS stakeholders (Specific aim 1) will review the adapted AAR tool until consensus on its applicability is reached.

#### Specific aim 3—interventions and comparisons

Consistent with the EPIS Implementation phase, the research team will pilot-test the AAR implementation strategy on improving CFT outcomes and explore team mechanisms. Specifically, to test the effectiveness of the AAR on improving outcomes associated with the CFT services intervention, we will implement randomization with parallel assignment in two CWS system regions, at the caseworker level. In each region, four CWS caseworkers will be followed as they engage in 5 CFT meetings (8 CWS caseworkers total). Within each region, two of the CWS caseworkers will be assigned to the team-enhanced implementation strategy, CFT + AAR (4 CWS caseworkers total), and two CWS caseworkers will be assigned to the standard implementation, CFT as usual (4 CWS caseworkers total). CFT as usual consists of CFT Meeting Facilitation wherein a mental health services organization partners with CWS to schedule the CFT meetings. The research team will utilize blocked randomization of CWS caseworker to intervention condition (CFT + AAR or CFT as usual) to ensure equal group sizes. As such, assignment of condition will be implemented by the research team without concealment or blinding.

Child mental health concerns are identified by a preliminary administration of the Child & Adolescent Needs & Strengths (CANS) [55] assessment for children aged 6–17 by the CWS caseworker at the initial family visit, which typically occurs 2–3 weeks prior to the initial CFT meeting. Based upon this initial visit and preliminary CANS results, informal and formal support persons are invited to the CFT meeting by the CFT Meeting Facilitation program. The CANS results are an integral component of the initial CFT meeting, during which the CWS caseworker guides discussion around the mental health needs that emerged from the initial CANS assessment and finalizes the Action Plan to attend to these needs. In addition to the above, those in the CFT + AAR will be guided through the AAR team effectiveness intervention, as facilitated by research staff, immediately following the CFT meeting. To optimize assessment of mental health services linkage, only CFTs which include a child with an identified mental health service determined by the preliminary administration of the CANS assessment by the CWS caseworker at the initial family visit will be included.

**Fig. 2:**
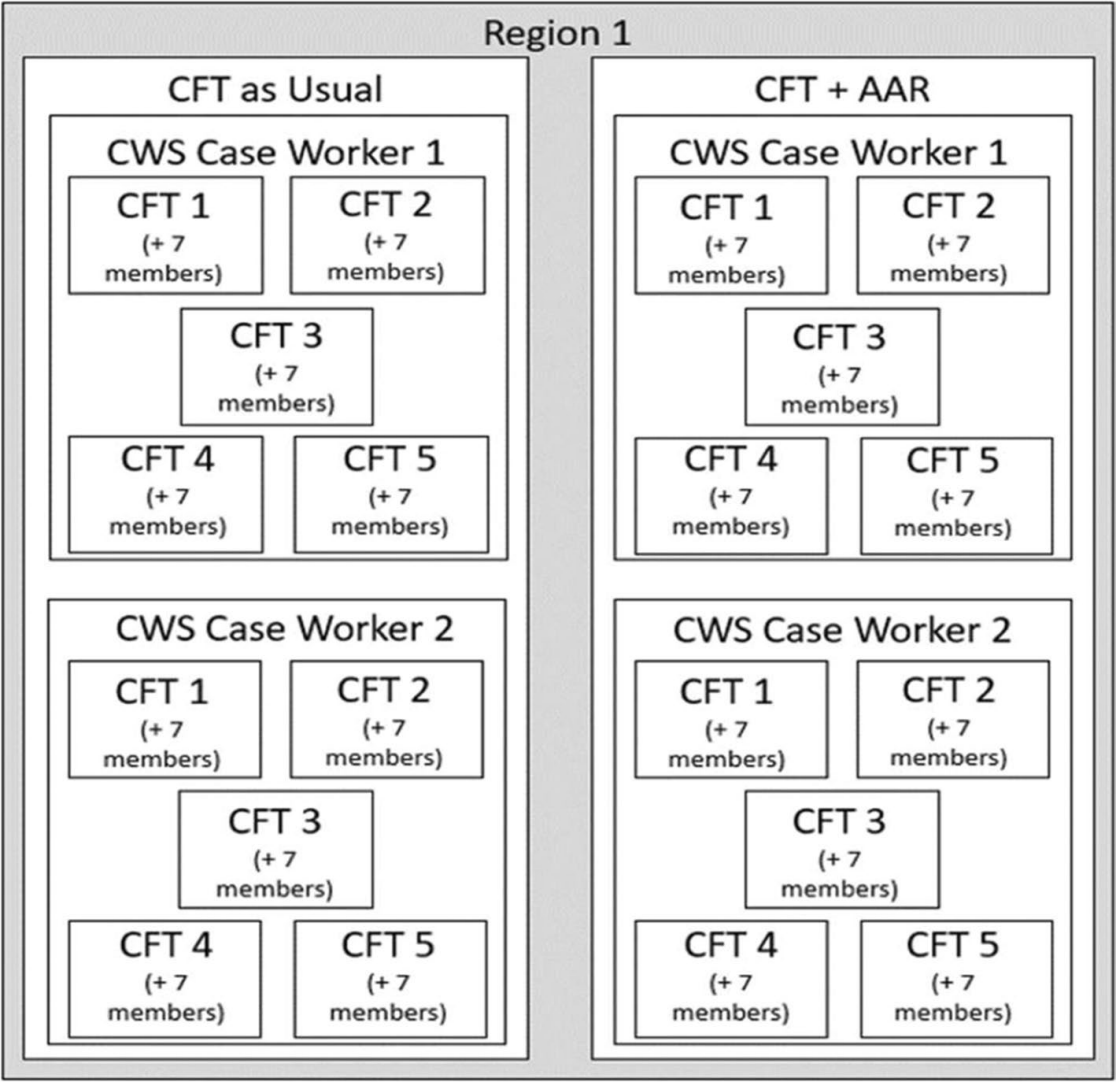
Participant sampling strategy. *Note.* Participant sampling by team, CWS caseworker, and condition. In total, 320 assessments will be collected from project participants from two County regions—8 team members from 8 child-family teams (4 per region) will participate in data collection following each of 5 CFT meetings. Half will be assigned to the control, CFT as usual, and the other half will be assigned to the intervention, CFT + AAR. Sampling is mirrored for Region 2

Immediately upon completion of each of the 5 CFT or CFT + AAR team meetings, the CWS caseworkers together with ~ 7 additional CFT members will provide data a via web-based online survey to assess mechanisms and outcomes. This results in 8 caseworkers leading 5 CFTs each for a total of 40 unique CFTs. Each of the 40 CFTs has ~8 participants, for a minimum total of 320 data points. See Fig. 2 for sampling.

### Setting and context

The CWS system under study operates across six County regions. In the 2019/2020 fiscal year, 4080 children ages 6–17 were referred to CWS per month, with an average of 1488 open cases each month, and an average of 1026 children 6–17 years in out-of-home each month. Compared to the 2020 child population of County (*n* = 760,789), a greater percentage of children in out-of-home care were Hispanic/Latinx (50% vs 42%) and Black (18% vs. 5%). A lesser percentage of CWS children were White (26% vs 37%), and Asian/Pacific Islander (3% vs. 11%). Majority of CWS referrals are for general neglect (42%), emotional abuse (33%), physical abuse (31%), at risk/sibling abused (21%), and sexual abuse (18%) [56]. CWS caseworkers are typically female (83%) and Hispanic/Latinx (44%).

### Participants

There are four types of participants in this study. Participants will be (1) County CWS system leaders and caseworkers, (2) CFT informal and formal meeting members, (3) parents/caregivers with open CWS cases, and (4) children aged 6–17 with open CWS case. CWS caseworkers and CFT informal and formal support persons will be ≥ 18 years of age. Children and family members urrently open to CWS have a minimum age eligibility of 6 years to correspond with the CWS protocol for screening for behavioral health services need, and no maximum age limit for eligibility. Participants will be recruited from select regions, which will be determined in collaboration with CWS leadership prior to project implementation. Inclusion criteria for all groups of participants are intentionally broad to ensure valid analyses.

The research team will partner with County CWS to identify appropriate and representative CWS caseworkers to follow over the course of 5 CFT meetings. The 5 CFT meetings per CWS caseworker will be identified to best represent County CWS open cases. Additionally, only CFTs which include a child with an identified behavioral health services need will be included to optimize assessment of behavioral health services linkage. Behavioral health service need is determined by a preliminary administration of the Child & Adolescent Needs and Strengths (CANS) assessment by the CWS caseworker at the initial family visit. The CANS behavioral and emotional needs domain is employed for all children aged 6–17 as the screener for behavioral health services.

For children aged 6–17 open to CWS and with mental health services needs (identified via CANS screening and confirmed during initial CFT meeting perusual CWS), County Behavioral Health administrative data will be extracted to determine mental health service linkage. While children aged 6–17 may also participate in the CFT meeting, this is considered part of usual CWS services. No additional study participation from these children will be solicited. The proposed study will not apply any gender or minority exclusion criteria for participant selection. All efforts will be made to ensure that women and minority representation will be representative of County CWS.

### Measures

Several measures will be administered to participants to assess mechanisms and outcomes of the AAR. See Table 1 for a list of measures to be administered to intervention participants, and proposed mechanism and/or outcomes assessed by each measure.

#### Acceptability, feasibility, and appropriateness of intervention measure

This 9-item measure [51] will be administered to CFT + AAR participants immediately following completion of the CFT + AAR intervention to assess the extent to which the after-action review is appealing, liked, and welcomed in their setting (acceptability); fitting, suitable, and applicable in their setting (appropriateness); and possible and doable in their setting (feasible). Each of the 9 items is rated using the following response options: 1 = completely disagree, 2 = disagree, 3 = neither agree nor disagree, 4 = agree, and 5 = completely agree. Three subscales can be created by averaging responses for items representing acceptability, appropriateness, and feasibility, respectively.

#### Card-sorting task

Card sorting elicits individual mental models to understand how participants structure their knowledge. In open card sorts, participants are provided with a set of key concepts and asked to sort them into categories. Each participant then creates a label for each category. In closed card sorts, participants are similarly given a set of key concepts, but participants are given pre-defined categories that each concept must be sorted into. Within team environments, card sorting can be used to examine the degree to which team members are thinking about key concepts in a similar manner (e.g., team mental models) by comparing the categories and sorted content across members. Each participant will complete the card-sorting task immediately following completion of the intervention and will assess CFT team mental models by examining perceptions of member roles, responsibilities, and goals across CFT team members. In doing so, the concepts contained in the card sort will be representative of specific CFT roles, task responsibilities, and goals.

#### Collaboration & Satisfaction About Care Decisions (CSACD)

The 6-item collaboration subscale [57] assesses collaboration, shared responsibilities for planning, open communication, and coordination. The 3-item satisfaction subscale assesses satisfaction with the decision-making process, and satisfaction with the decision itself [57]. Although originally developed about care decisions for intensive care patients, it has since been used to measure collaboration and satisfaction with treatment planning plans associated with after-action reviews. Each participant will complete the CSACD immediately following their completion of the intervention. Participants respond on a 7-point Likert-type scale ranging from 1 = strongly disagree to 7 = strongly agree. Subscale scores are computed by calculating the average item response for items representing collaboration, and items representing satisfaction, respectively.

#### Demographics

Participant age, gender, race, ethnicity, marital status, education level, primary language, and working status/profession will be collected of all participants immediately following completion of the intervention.

#### McAllister affect-based trust

This 5-item scale [58] assesses perceptions of affect-based trust amongst CFT members. Items center on individual’s perceptions that team members can share ideas freely, that team members listen to one another, that team members care for one another, and that team members are invested in working well with one another. Each participant will complete this measure immediately following their completion of the intervention. Participants respond on a scale ranging from 1 = strongly disagree to 7 = strongly agree, and scale scores are computed by calculating the average item response.

#### Parent Participation Engagement Measure (PPEM)

This 5-item measure originally developed for culturally diverse families served in children’s outpatient mental health, was adapted for CWS home visitation services, and assesses active parent participation, engagement, and voice in treatment services [59]. Each participant will complete the PPEM immediately following their completion of the intervention. Participants respond on a 5-point Likert-type scale ranging from 1 = not at all to 5 = very much. Scale scores are computed by calculating the average item response.

#### Psychological safety

Edmondson’s 7-item Psychological Safety Climate Measure [41] evaluates perceptions of the work environment (in this case, the CFT meeting) as being one wherein policies and procedures foster a safe and comfortable space for interpersonal risk. Each participant will complete the Psychological Safety Climate Measure immediately following their completion of the intervention. Participants respond on a scale ranging from 0 = doesn’t apply at all to 4 = entirely applies, and overall scale scores are computed by calculating the average item response.

#### Working Alliance Inventory Short Form (WAI-SR)

The WAI-SR [60] is a 12-item version of the Horvath and Greenberg’s (1989) Working Alliance Inventory. Each item is responded to using a 7-point Likert-type scale. It yields three summed subscale scores, as well as one summed overall scale score. Each of the subscales will measure proposed mechanisms, while the overall scale will measure proposed outcomes. The 4-item Goal subscale will be used to determine team member agreement on goals for CWS child/family case treatment plan (goal specification). The 4-item Task subscale will be used to determine team member agreement on how to achieve goals for CWS child/family case treatment plan (strategy formulation). The 4-item Bond subscale will be used to determine the personal bond between CFT team members (trust). The overall scale score assesses the collaborative relationship between relevant parties and consensus and willingness by all to engage in and do the work that leads to improvement (CFT Fidelity). Each participant will complete the WAI-SR immediately following their completion of the intervention.

### Data management

All collected data will use codes rather than subject names. Only the primary research team members will have access to participant identifiers. The research team will use a university-approved data entry, training, and management system that complies with the data security requirements of the Health Insurance Portability and Accountability Act of 1996 (HIPAA) [61] and the Health Information Technology for Economic and Clinical Health (HITECH) Act [62]. Aim 1 qualitative interview data will be de-identified and transcribed by study personnel or HIPAA compliant transcription services. Survey data for Aim 3 will be collected electronically at the end of the CFT meeting using secure online survey program software, with individual distribution links shared at the end of the CFT meeting. Web-survey data and qualitative transcripts kept by the research project will be coded with a unique identifying number for which the key will be separately stored and limited to primary research staff members who will only share these data to the extent necessary for required reporting purposes. Audio recording data from interviews and focus groups will be uploaded by study staff securely to the HIPAA compliant server.

### Analytic plan

Multilevel modeling with random intercepts and slopes will be used to examine differences in the targeted outcomes between CFT and CFT + AAR conditions, while accounting for the nested data structure. Specifically, this project will test the main effect of the AAR on implementation outcomes and child/family outcomes. Analyses will include the between-CFT meeting factor of condition (CFT vs. CFT + AAR) and within-CFT meeting factor of individual CFT member participant. The cross-level condition by participant interaction effect on the specified outcomes will be of primary interest.

A cross-level prediction model will test the extent to which the condition effect on the CFT outcomes is mediated by its proximal effect on team processes and emergent states. To assess the extent to which team processes are targeted by the AAR intervention, the Goal and Task subscales of the Working Alliance Inventory-Short Revised (WAI-SR) will be used to assess goal specification and strategy formulation, respectively [60, 63]. The Collaboration subscale of the Collaboration & Satisfaction About Care Decisions (CSACD) [57] measure will be used to assess the extent to which the AAR engages the team processes of communication and coordination. Several measures will be used to assess the extent to which emergent states are targeted by the AAR. Specifically, the Bond subscale of the WAI-SR and the McAllister Affect-Based Trust [58] measure will be used to assess trust. Edmondson’s Psychological Safety Climate [41] measure will assess the extent to which the AAR impacts the emergent state of psychological safety. To assess the extent to which the AAR engages the emergent state of shared mental models, a card-sorting task will assess CFT team shared mental models by examining perceptions of member roles, responsibilities, and goals across CFT team members. The concepts contained in the card sort will be representative of specific CFT roles, task responsibilities, and goals. To determine the feasibility, acceptability, and appropriateness of the AAR for CWS CFTs, those in the CFT + AAR condition will complete the Feasibility, Acceptability, and Appropriateness of Intervention Measures, respectively [51].

### Power analysis

Based upon the sampling strategy, power analysis was conducted to inform the detectable magnitude of effect. Power for the primary CFT services intervention outcomes, and team processes/emergent states outcomes, was estimated using a multi-step approach recommended by Hox [64]. First*,* a total number of observations was computed while ignoring nesting. With 8 team members from 8 child-family teams (4 per region) participating in data collection following each of 5 child-family team meetings, there is a total of 320 observation results. For these observations, the nested data structure introduces dependency that decreases the level of statistical power. To adjust for this, a second step was to penalize the total sample for nesting. This was accomplished using a reorganization of the design effect formula (i.e., *n*_*eff*_ = *n*/[1 + [*n*_*clus*_ − 1]*ρ*]), which provides the effective sample size of independent observations. Using conservative nesting effects for CFT members within CFT meetings, *ρ* = 0.20, the 320 observations provide statistical power equivalent to 36 independent observations. Third, the effective sample size was used to perform conventional, single-level power analysis using GPower. Assuming power of 0.80 and *α* = 0.05, results indicated that a small-to-medium main effect of *f*^2^ = 0.10 for condition on CFT clinical intervention outcomes may be detected. As noted, for the preliminary mediation tests, statistical power is necessarily limited. Simulations performed by Fritz and MacKinnon [65] have demonstrated that required sample sizes for mediated effects range from 35 to 74. This depends on the magnitude of the effects for the respective paths, with the smaller sample sufficient if both paths have large effects and the larger sample sufficient if both paths have medium effects. As such, the effective sample size of 36 is potentially sufficient if the magnitude of each effect for the respective paths is great.

### Dissemination plan

Beyond plans to submit data elements to ClinicalTrials.gov, aggregate data will be synthesized and summative feedback of the findings and areas that warrant further attention will be provided to CWS. This will provide practically relevant and actionable data to inform policy and practice implementation locally. Additionally, study findings will be prepared and submitted for publication in peer-reviewed scientific journals. All peer-reviewed scholarly publications will be submitted to the National Center for Biotechnology Information and PubMed Central at the US National Institutes of Health’s National Library of Medicine.

## Discussion

Effective CWS practice relies on the role of child protection caseworker in partnering with children and families to create a service Action Plan directed towards family strengths and needs. This study responds to the critical importance of including families in decision-making processes, which facilitates engagement in the Action Plan, promoting use of needed behavioral health services. This study has the potential to demonstrate the feasibility and utility of the AAR to enhance CFT implementation and effectiveness.

As with any research study, the potential for operational issues is present and may serve as a challenge to performing the study. The COVID-19 pandemic has intensified disparities for several communities [66]. In the event that CFT meetings have not resumed in person, the research team may encounter related practical challenges through the use of a virtual meeting platform. Given that communities may have unequal access to virtual resources [66], including cell phones and laptops to stay engaged with services, there may be a limited range of sample that lacks economic and/or racial diversity. In addition to resource inequity and restricted range of sample, there could be additional, unanticipated technological barriers when conducting the AAR team effectiveness intervention following the virtual CFT meetings.

The research team remains sensitive to the potential coercion of parent/caregiver—an issue that is discussed often in the context of CWS participation for which parents/caregivers may feel obligated to “demonstrate cooperation [67]”. To the best of the ability of the research team, this concern for potential coercion as specifically related to the research is mitigated by several intentional considerations. First, the informed consent process will be conducted by a member of the research team who has completed a training in cultural competence in research and evaluation. Then, the informed consent process itself includes multiple components. Introductions to the relevant individuals will clearly identify the researchers, who will define the purpose and nature of the study and ensure that parent/caregiver understands the scope of what is being asked of them. Prospective parent/caregiver will have an opportunity to ask questions before deciding to participate. Objectives will be made clear. An honest assessment of the risks or potential adverse impacts of the research, which should be minimal for these participants, will be provided. Potential usefulness of the research for the child-welfare involved children and parents/caregivers in general will also be explained. Privacy and confidentiality will be addressed throughout the study.

The research team, and all consent forms, will underscore that enrollment and continued participation are completely voluntary. And, of utmost importance, the research team will ensure that potential participants fully understand that their declination to participate in the research component will in no way negatively impact the services that they or their children will receive as part of their child welfare involvement. Parents/caregivers may terminate participation at any time if they become uncomfortable with the research study. Participants will be provided with investigator contact information if they wish to discuss concerns about discomfort. Also, prior to enrolling any participant, a clear understanding will be documented from CWS administration that the study will not be able to share any individual data with CWS, and that no individual’s study data will be used for CWS-related service provision. As an additional safeguard against coercion, CWS caseworkers will be naïve to whether parents/caregivers consent or decline research participation.

This project has the potential to benefit service delivery and integration for CWS leaders, caseworkers, formal and informal CFT member support persons, parents/caregivers, and children with open cases. By inclusion of child and family voice, the AAR-enhanced CFT should lead to increased fidelity to the CFT intervention and greater levels of parental satisfaction and shared decision-making, thus resulting in enhanced follow-through with service plans and linkage to mental health treatment services for children. Improving intervention effectiveness, both at the system and family levels, is crucial for practice efficiencies and improved child and family outcomes.

## Data Availability

N/A.
